# Genome Sequence of the *Sulfitobacter* sp. Strain 2047-Infecting Lytic Phage ΦCB2047-B

**DOI:** 10.1128/genomeA.00945-13

**Published:** 2014-01-16

**Authors:** Nana Y. D. Ankrah, Charles R. Budinoff, William H. Wilson, Steven W. Wilhelm, Alison Buchan

**Affiliations:** a Department of Microbiology, University of Tennessee, Knoxville, Tennessee, USA; b Bigelow Laboratory for Ocean Sciences, East Boothbay, Maine, USA

## Abstract

We announce the complete genome sequence of a lytic podovirus, ΦCB2047-B, which infects the bacterium *Sulfitobacter* sp. strain 2047, a member of the *Roseobacter* clade. Genome analysis revealed ΦCB2047-B to be an N4-like phage, with its genome having high nucleotide similarity to other N4-like roseophage genomes.

## GENOME ANNOUNCEMENT

Bacteria of the *Roseobacter* lineage, which includes the genus *Sulfitobacter*, are abundant marine heterotrophs that mediate several key biogeochemical processes, including the transformation of organic and inorganic sulfur compounds, the oxidation of carbon monoxide, and the degradation of vascular plant material (1–3). Roseobacters, along with their infecting phages, are excellent models for studying how microbial activities shape biogeochemical cycles (4). Here, we report the genome sequence of phage ΦCB2047-B, which infects *Sulfitobacter* sp. strain 2047.

The phage was isolated from a mesocosm study in Raunefjorden, Norway, using standard virus enrichment and plaque assay techniques (4, 5). Phage DNA was submitted to the Broad Institute and sequenced under the Gordon and Betty Moore Foundation’s Marine Phage, Virus, and Virome Sequencing Project. The Broad Institute sequencing data were assembled using the Lasergene SeqMan Pro. The assemblies resulted in the generation of a single contig, which had sequencing coverage of approximately 30×. The contig was annotated using RAST and the tRNAscan-SE search server (6, 7). Translated peptides from the phage genome were used as BLASTp queries to the NCBI nonredundant protein sequence database to manually curate possible gene functions and to identify the nearest phage or prophage relatives. The CoreGenesUniqueGenes (CGUG) genome analysis tool (8) was used to identify gene homologues and assign core genes shared with other N4-like phages.

Phage ΦCB2047-B is 74,480 bp (74.5 kb) with a G+C content of 43% and 92 identified open reading frames. The genome sequence indicates this is an N4-like bacteriophage that is highly similar to but genetically distinct from other recently described roseophages (9). Morphological analysis by transmission electron microscopy confirmed that phage ΦCB2047-B belongs to the family *Podoviridae*. The genome content and architecture of ΦCB2047-B are similar to those of other N4 phages. Consistent with most other N4-like phages, the genome possesses 437-bp direct terminal repeat sequences on its distal ends. A CGUG analysis identified 20 highly homologous genes (BLASTp threshold score, 85) between phage ΦCB2047-B and these previously reported N4-like phages: the enterobacterium phage N4 (accession no. NC_008720), *Pseudomonas* sp. phages LUZ7 (accession no. FN422398) and LIT1 (accession no. FN422399), and N4-like roseophages ΦDSS3P2 (accession no. FJ591093) and ΦEE36P1 (accession no. FJ591094). An analysis focused exclusively on N4-like roseophages (ΦDSS3P2 and ΦEE36P1) identified 41 genes with high homology. Genome-wide nucleotide similarity alignments with the ΦDSS3P2 and ΦEE36P1 genomes showed that phage ΦCB2047-B shares 43.9 and 44.4% nucleotide identity, respectively. Unlike other N4-like phages, ΦCB2047-B contains a deoxycytidine triphosphate (dCTP) deaminase instead of a deoxycytidine monophosphate deaminase, indicating a preference for an alternative route for the generation of dUMP for thymidine biosynthesis. The closest homolog in the NCBI database to the phage dCTP deaminase is from a coliphage, EC1-UPM (accession no. AGC31535), which has 37% identity. The host genome also contains a homolog to this protein that shares 29% identity to the phage gene and suggests genetic divergence.

### Nucleotide sequence accession number.

The complete sequence of the *Sulfitobacter* phage ΦCB2047-B genome can be accessed under the GenBank accession no. HQ317387.
